# Desert Microbes for Boosting Sustainable Agriculture in Extreme Environments

**DOI:** 10.3389/fmicb.2020.01666

**Published:** 2020-07-22

**Authors:** Wiam Alsharif, Maged M. Saad, Heribert Hirt

**Affiliations:** ^1^DARWIN21, Biological and Environmental Sciences and Engineering Division, King Abdullah University of Science and Technology (KAUST), Thuwal, Saudi Arabia; ^2^Max Perutz Laboratories, University of Vienna, Vienna, Austria

**Keywords:** PGPR, plant microbiota, desert agriculture, world hunger, desert microbes, DARWIN21

## Abstract

A large portion of the earth’s surface consists of arid, semi-arid and hyper-arid lands. Life in these regions is profoundly challenged by harsh environmental conditions of water limitation, high levels of solar radiation and temperature fluctuations, along with soil salinity and nutrient deficiency, which have serious consequences on plant growth and survival. In recent years, plants that grow in such extreme environments and their naturally associated beneficial microbes have attracted increased interest. The rhizosphere, rhizosheath, endosphere, and phyllosphere of desert plants display a perfect niche for isolating novel microbes. They are well adapted to extreme environments and offer an unexploited reservoir for bio-fertilizers and bio-control agents against a wide range of abiotic and biotic stresses that endanger diverse agricultural ecosystems. Their properties can be used to improve soil fertility, increase plant tolerance to various environmental stresses and crop productivity as well as benefit human health and provide enough food for a growing human population in an environment-friendly manner. Several initiatives were launched to discover the possibility of using beneficial microbes. In this review, we will be describing the efforts to explore the bacterial diversity associated with desert plants in the arid, semi-arid, and hyper-arid regions, highlighting the latest discoveries and applications of plant growth promoting bacteria from the most studied deserts around the world.

## Deserts Cover One-Third of the Earth’s Land Surface

**FIGURE 1:**
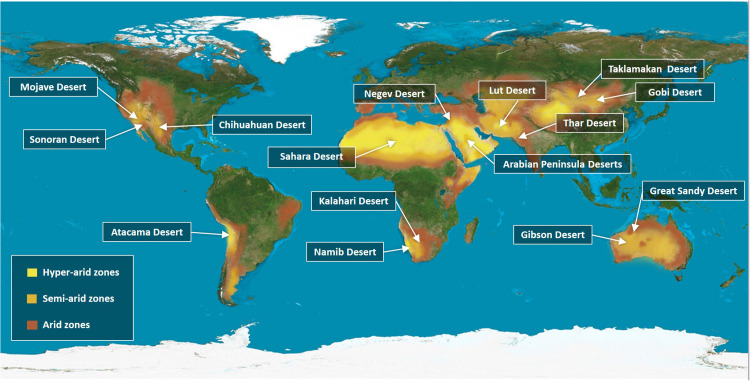
A global distribution of the desert areas according to their Aridity index. Deserts distribution throughout the land surface, making up 33% of the total land area. The map indicates the most famous deserts in each continent, the Mojave, Sonoran, and Chihuahua Desert in North America and the Atacama Desert in South America. The largest desert are in the world the Sahara Desert in North Africa, along with the Kalahari and Namibia deserts in the southern parts of Africa. In the Middle East, the Negev and the Arabian Peninsula Deserts. In East Asia, the Thar Desert in North India and parts of Pakistan and in west Asia the Lut Desert, while the Gobi and Taklamakan Deserts cover North China and a large part of Mongolia. In Australia, Great Sandy and Gibson Deserts are shown. The map classifies the deserts based on the global Aridity index (AI), which is defined as the numerical indicator of the degree of climatic dryness in a specific location. The map shows the hyper-arid zones in bright yellow, semi-arid zones in orange, and arid zones in brown. Source: Adapted from World Atlas of Desertification (Cherlet et al., 2018).

The Earth’s surface is covered by 71% of water and 29% of land. Deserts are found in every continent and they make up 33% of the total land area (Figure 1). In this review, we are covering some of the major representative deserts that have been extensively studied over the past years, such as the Kalahari and Namib deserts in South Africa and the largest desert in the world, the Sahara in North Africa (Goudie, 2009). In the Middle East, the Arabian Peninsula embraces one of the largest sandy deserts in the globe, namely the Empty Quarter (Edgell, 2006) and the Negev (Kaplan et al., 2013). In Asia, the Gobi is the world’s highest and driest desert that covers North China and a great part of Mongolia. Bound to the Gobi desert from the West is the Taklamakan Desert (Rosen et al., 2019). In North India and parts of Pakistan, the Thar is one of the most heavily populated deserts (Ramawat, 2010; Rao et al., 2016). The Mojave, Sonora and the Chihuahua deserts are located in North America, while the Atacama is located in South America. Finally, deserts in Australia cover up to 40% of the land area, with the Simpson in the North and the Gibson and Great Sandy deserts in the West. Given that deserts cover one-third of Earth’s land surface, exploiting these vast, dry and isolated landscapes for agriculture will help us overcome some challenges of achieving global food security for the overgrowing human population.

## Deserts Encompass Extreme Environmental Conditions

Life in these regions is profoundly challenged by the harsh abiotic stresses, which negatively impact the living organisms (e.g., plants and microbes) by the non-living stressors such as drought, salinity, low or high temperatures, and other environmental extremes. Extreme temperature fluctuations are one of the main features associated with most deserts, at daytime the sun can rapidly heat up the desert due to the low vegetation coverage, while at night an abrupt drop in temperatures can occur, it has been reported that in some of the hot deserts the daytime temperature can range between 40 and 50°C and drop down to 0°C in the night (Ramawat, 2010).

High solar radiation in the desert is another environmental challenge, where radiation can be as high as 840 GJ km^2^ year^–1^ (Goudie, 2010). The area extending from the Arabian Peninsula to the Sahara is one of the largest areas receiving the highest radiation levels in the world (Edgell, 2006; Goudie, 2010). The main factors behind this high radiation levels are the proximity of these deserts to the equator and the low cloud coverage that allows high amounts of radiation to reach the Earth’s surface (Greenville, 2018).

Water scarcity is another major abiotic stressor in desert habitats, as deserts are considered to be water-controlled ecosystems, where survival can be maintained through the balance between water loss and water availability (Flanagan and Ehleringer, 1991). Thereby, the main natural sources of water for life in the deserts are precipitation (e.g., rainfall), surface flow, groundwater, atmospheric vapor and fog (Flanagan and Ehleringer, 1991; Zangvil, 1996; Wang et al., 2017). In addition, low annual rainfall combined with high evaporation rates due to high temperatures results in rapid reduction of water reserves in deserts (Ceballos et al., 2004).

The soil in desert regions is extremely arid due to several factors: strong wind erosion, sedimentation, daily temperature fluctuations and, most importantly, water deficiency. In most desert regions, the soil is characterized as dry “Aridisols” with very low nitrogen content and organic matter, slightly alkaline pH, high salt ions content, higher phosphate, calcium carbonate, and magnesium carbonate contents in all or some of the soil parts (Archibold, 1995; McHugh et al., 2017). Furthermore, one of the most distinguishing characteristics of dry soils with sparse vegetation cover such as desert soils, is the biological soil crusts (BSCs). BSCs are composed of soil particles mixed with filamentous cyanobacteria, lichens, mosses, and fungi (soil-surface community) in a cohesive manner that cover the soil surface and maintain the soil fertility and stability in harsh infertile desert ecosystems (Belnap, 2003). The cohesive nature of BSCs allows it to stabilize the soil by protecting it from water and wind erosion and to enhance the soil physical structure by forming soil aggregates. BSCs also help nourishing the soil by capturing the nutrient rich dust with essential nutrients (e.g., nitrogen, potassium, and phosphorus) and improve the water holding capacity of the soil (Belnap et al., 2003; Faist et al., 2017). This cohesiveness is due to the formation of a polysaccharide sheath on the soil surface which is produced by some of the BSC microbial community members (e.g., filamentous cyanobacteria) (Reynolds et al., 2001; Belnap et al., 2003; Williams et al., 2012; Richer et al., 2015; Weber et al., 2015; Faist et al., 2017).

Biological soil crust communities are essential contributors in nourishing the dry environments with nutrients. For example, carbon and nitrogen are fixed from the atmosphere by microbes and released into the dry surrounding soils in forms that can be utilized by other organisms such as nearby plants, bacteria, and fungi. Therefore, BSCs contribute to soil fertility, thereby increasing microbial biomass and enhancing plant growth (Belnap et al., 2003; Weber et al., 2015).

Another feature of desert soils is soil salinity, saline soils represent a major problem that threat the desert environments, it has been reported that about 20–30% of the global irrigated lands are salt affected, and around 50% of the global arable lands are estimated to be salinized by 2050 (Whitford and Wade, 2002; Qadir et al., 2014; Shrivastava and Kumar, 2015). Additionally, the accumulation of salt ions in the soil occurs due to high evaporation rates and low precipitation levels, and saline soils have severe consequences of osmotic stress, ion toxicity, and imbalance for the plants dwelling in the deserts. Excessive amounts of sodium (Na^+^) and chloride (Cl^–^) ions have negative consequences on plant membranes and enzymes disturbing energy balance and protein metabolism (Shrivastava and Kumar, 2015).

## Adaptation of Desert Plants to Extreme Environmental Stresses

Despite the multitude and severity of environmental challenges found in deserts, plants, animals and microbes have developed adaptation mechanisms that allow their growth, survival, and reproduction under these extreme conditions. Deserts were home to many old civilizations in the Sonoran, Mojave, Chihuahua, and Atacama deserts and in the Middle East (Pritzker, 2000). Early civilizations that lived in these deserts used plants for food and feed, clothes, shelter, fuel, and medical remedies. Today, plants serve an important role in providing economically, medically and industrially beneficial values to humans (Table 1). The extreme environmental conditions of the desert habitats result in scarce plant growth, limiting the vegetation of deserts mostly to shrubs and grasses. The flora of deserts mostly consists of short-lived annual plants or succulent perennial plants (stem or leaf succulents) adapted to conserve the moisture and non-succulent perennials that include shrubs, herbs, grasses, and trees (Monson, 2014).

**TABLE 1:** Summary of famous desert plant species found within the global deserts and some of their beneficial values.

Location	Desert	Plant species	Beneficial values (medical and economic importance)	References
**Africa**	**The Sahara Desert**	**Perennial shrubs**: *Ephedra alata* **Trees**: *Tamarix aphylla* (Athel tree) **Halophytes**: *Atriplex* (Saltbush) *Salsola* (Saltwort) **Grasses**: *Aristida plumose*	• *E. alata* stems produce alkaloid ephedrine that serve medical purposes to treat asthma, the fibrous stem and roots of *E. alata* are used for making cords, twines, and strings. • *T. aphylla* has special fire adaptability due to its high salt content, which makes it used as a fire • The blossoms of *T. aphylla* produce high quality honey. • *Salsola soda* are used for cooking as a vegetable in Italy.	Archibold (1995), Hegazi and Ellamey (2011)
	**The Namib Desert**	**Shrubs**: *Acanthosicyos horrida* (Nara) **Flowering plants:** *Welwitschia mirabilis* (Tumboa) **Grasses**: *Stipagrostis* sp. **Trees**: *Colophospermum mopane* (Mopane) **Succulents**: *Conophytum Lithops* (Living stone) *Pachypodium* **Trees**: *Aloidendron pillansii* (Giant quiver tree) *Portulacaria afra* (Elephant bush)	• *Acanthosicyos* horrida is an endemic fruit only found in the Namib and the Nama people consume it as a fruit. • *C. mopane* trees used for several wood production purposes (decorative wood, floors, musical instruments, essential oils, etc.) • *P. afra* is used for ornamental purposes (e.g., as a Bonsai tree), and it is a common food in South Africa.	Hartmann (2002); Oliveira and Koptur (2017)
	**The Kalahari Desert**	**Grasses**: *Stipagrostis amabalis* **Trees**: *Acacia erioloba* (Giraffe thorn) *Terminalia sericea* (Silver Terminalia)	• *T. sericea* leaves and roots are used as a traditional medicine to treat pneumonia, bilharzia and as a stomach remedy. *T. sericea* leaves and roots are used as a traditional medicine to treat pneumonia, bilharzia and as a stomach remedy. *T. sericea* leaves and roots are used as a traditional medicine to treat pneumonia, bilharzia and as a stomach remedy.	Palgrave et al. (2002)
**Australia**		**Grasses**: *Triodia basedowii* (Lobed spinifex) *Astrebla* (Mitchell grass) **Trees**: *Eucalyptus brevifolia* (White gum) *Acacia aneura* (Mulga) *Eucalyptus* (Eucalypts) **Halophytes**: *Atriplex* (Orache) *Maireana*	• *Eucalyptus* Trees are used for several purposes such as production of honey, Eucalyptus oil that is used in the industry of making (cough drops, toothpaste, food supplements, insect’s repellent, etc.).	Clarke (2012)
**Asia**	**The Gobi Desert**	**Trees**: *Ulmus pumila* (Siberian elm) **Shrubs**: *Anabasis brevifolia Tamarix ramosissima* (Salt cedar) *Haloxylon ammodendron* (Saxaul tree) **Grasses**: *Aneurolepidium* (Giant wildrye) *Artemisia annua* (Sweet wormwood)	• In China, *H. ammodendron* is used as a preventive management tool against desertification and for stabilization of the sand dunes due to its high drought-resistant characteristic. • *Artemisia annua* produces *Artemisinin* which is used in malaria treating drugs, and *A. austriaca* helps with morphine withdrawal.	White (1997)
**North America**	**The Sonoran Desert**	**Succulents**: *Cereus giganteus* (Giant Cactus or Saguaro) *Opuntia* (Prickly pear) **Subtrees**: *Cercidium microphyllum* (Yellow paloverde) **Legumes**: *Prosopsis glandulosa* (Honey mesquite) *Olneya tesota* (Desert ironwood) **Shrubs**: *Ambrosia dumosa* (White bursage) *Fouquieria splendens* (Ocotillo) *Larrea tridentate* (creosote bush)	• Native Americans used *Cereus giganteus* for food, building material, it is also used by birds as nests by creating holes in the cactus. • *Opuntia* fruits are used for food by the locals, it also produces several chemicals with medical properties such as polyphenols, betalains, gallic acids and vanillic acid, and they are also used in dye production. • *Cercidium microphyllum* was used by the Native Americans as flours, food, and jewelry. • *Prosopsis glandulosa* is known as honey mosquite which is due to its honey production that supports the plant pollinator species such as bees.	Guzmán-Maldonado et al. (2010)
	**The Chihuahuan Desert**	**Shrubs**: *Agave Flourensia cernua Acacia constricta*	• The sap of *Agave americana* is used in Mexico to produce alcoholic beverages, and in the young flower stalks a nectar can be extracted and used as a natural sweetener in cooking.	
	**The Mojave Desert**	**Trees**: *Yucca brevifolia* (Yucca palm) Flowering plants: *Grayia spinosa* (Hop sage) *Chilopsis linearis* (Desert-willow) *Ceratoides lanata* (winterfat) **Grasses**: *Agropyron* (Wheatgrass)	• Yucca trees were used by Native Americans to make sandals, baskets, dye, and food. • Chilopsis plants are used as a traditional medicine for fungal infections (e.g., athlete’s foot), cough, and wounds.	Zacharias and Baldwin (2010)
**South America**	**The Atacama Desert**	**Trees**: *Prosopsis juliflora* **Grasses**: *Distichlis spicata* (Desert saltgrass) *Sporobolus virginicus* (sand couch grass) *Succulent halophytes*: *Salicornia fruticose* (Glasswort) **Shrubs**: *Capparis angulate* (Caper shrub) **Succulents**: *Neoraimondia gigantean Haageocereus repens*	• The main uses of *Prosopsis* plants are for animal forage and wood production; it produces an unusual amount of flavonol in its heartwood. Its disadvantage is its contribution in transmitting malaria. • Indigenous people used to scrap the salt off the *Distichlis spicata* plant surface to make salt blocks, due to its salt secreting property.	Sirmah et al. (2009), Muller et al. (2017)
**Middle East**	**The Arabian peninsula**	**Perennial Shrubs**: *Celastraceae Haloxylon persicum* (Saxaul shrub) *Scrophularia hypericifolia Cornulacea Arabica* (Khat) *Ephedra alata* **Leguminosae**: *AbrusIndigofera* **Grasses**: *Panicum turgidum* **Halophytes:** *Halocnemum strobilaceum Haplophyllum tuberculatum Nitraria retusa* **Date palms:** *Phoenix dactylifera* **Shrubs**: *Retama raetam* (Retem)	• *Nitraria retusa* is a common food for camels and goats in the desert, it’s also used a salt source, its wood used for fuel. • *Haloxylon persicum* is a source of firewood. • The main pharmacoactive compounds in Khat leaves are phenylpropylamino alkaloids (S)-cathinone and (S)-cathine, which cause stimulating psychoactive. • *Haplophyllum tuberculatum* serves medical purposes as antioxidant, antimicrobial, cardiovascular, and anti-inflammatory. • Date palms are famous within the Middle East for the nutritious values of the palms and used as a source of food.	Edgell (2006), Sabry et al. (2016)
	**Negev Desert**	**Trees**: *Acacia raddiana* Shrubs: *Retama raetam* Flowering plants: *Zygophyllum dumosum*, *Reaumuria hirtell*	• *A. raddiana* has several economical uses in wood production (furniture, tools, wheels, etc.), production of Gum (Acacia/Arabic Gum) and in the production of grazing animal fodder.	Ward et al. (1993)

High temperatures and high radiation levels can have detrimental effects on plants, such as the production of reactive oxygen species (ROS), which damage DNA, proteins and membrane lipids (Akashi et al., 2008). The adaptation to these stress levels includes a change in the leaf reflectance and orientation, where vertical and steep leaf angles reduce light absorbance and heat accumulation (Gibson, 1996). Another mechanism to cope with excessive light and temperature is the presence of thick wax layers on the leaf surfaces (Holmes and Keiller, 2002) or the production of phenolic compounds (e.g., flavonoids) that act as “UV-absorbing sunscreen” (Caldwell et al., 1998; Julkunen-Tiitto et al., 2015).

Another important mechanism that desert plants use to tolerate high temperatures and radiation is altering the photosynthetic process, in order to reduce the absorbed excessive energy, desert plants use photorespiration to reduce oxygen to carbon dioxide (Kozaki and Takeba, 1996). Other alterations of the photosynthetic processes include the Crassulacean acid metabolism (CAM) used by many desert plants, as found in Agave (Agavaceae) and cacti (Cactaceae) (Batanouny, 2001; Ludwig, 2012; Greenville, 2018). In addition, heat tolerance is achieved by the expression of heat shock proteins (HSP) which allow correct protein folding at high temperatures and, thereby, help maintain the plasma membrane stability (Al-Whaibi and Mohamed, 2011; Zhu et al., 2018). The expression of small HSPs and high levels of transpiration found within the heat tolerant succulent *Agave tequilana* allow these plants to thrive in high temperatures in the arid zones of Mexico (Lujan et al., 2009).

Since water scarcity is a major threat to plants residing in desert habitats, plants employ several strategies in order to escape, tolerate or adapt to drought stress, for example, a common strategy used by annual plants is to keep their seeds dormant, when they only germinate and develop when the water is available (Smith et al., 1997; Norton et al., 2016). On the other hand, perennial plants avoid the periods of limited soil moisture by morphological adaptations, such as developing roots that extend deep into the soil (e.g., *Acacia eriololba* or *Boscia albirtunca* trees in South Africa) with up to 70 m long roots, and succulent plants (e.g., cacti) close their stomata and use CAM photosynthesis (Monson and Smith, 1982). Other plants maintain reduced levels of gas exchange by inducing stomatal closure at low water potentials (Smith et al., 1997). In addition, small leaf size, vertical leaf orientation, low xylem hydraulic conductance, and maintenance of tissue turgor by accumulation of solutes and high tissue elasticity are found in a number of plant species such as *Anabasis articulate* or *Zygophyllum dumosum* (Smith et al., 1997).

Another adaptive strategy used by desert plants, specifically desert grass species, is the formation of soil particles that physically adhere to the root system in a strong manner, that appear as soil aggregations attached firmly to the root surface, this trait is known as the plant rhizosheath (Brown et al., 2017; Pang et al., 2017). The first rhizosheath reports were from drought tolerant desert grasses in sandy dunes of the semi-arid regions (Volkens, 1887; Price, 1991; Pang et al., 2017). Over the years, many reports highlighted the formation of rhizosheaths by different crop plants, such as wheat, barley, and maize (Watt et al., 1994; Young, 1995; Haling et al., 2010; Brown et al., 2017). Rhizosheaths are formed under the influence of several factors: root hairs, mucilage secretion from both plant roots and soil microorganisms, soil properties (e.g., acidity, moisture, and carbon content) and, most importantly, water availability in the soil (Carminati, 2013; Pang et al., 2017; Rabbi et al., 2018). Furthermore, larger rhizosheaths and an increased number of root hairs, root hair length and density are observed under water stress conditions, which suggests that the formation of the rhizosheaths is used as an adaptive mechanism by plant roots to tolerate drought stress (Watt et al., 1994; North and Nobel, 1997; Liu et al., 2019). In addition to drought tolerance, rhizosheath formation correlates positively with increased nutrient uptake (Delhaize et al., 2012; James et al., 2016; Pang et al., 2017).

Based on their ability to tolerate salinity, plants are classified as Glycophytes (plants that are not adapted to tolerate salinity) and Halophytes (plants that can thrive in elevated salinity that can reach up to 200 mM NaCl) (Cheeseman, 2015). Desert halophytes have evolved several salinity stress tolerance mechanisms, such as changes in the root morphology by having longer tap roots to access humidity at higher depths within the soil (Cheeseman, 2015). Other mechanisms involve the ability to sequester ions in granular compartments and vacuoles and the accumulation of compatible solutes (e.g., proline) for osmotic adjustment and the maintenance of cell turgor (Munns and Tester, 2008). In addition, desert plants use mechanisms to limit root salt uptake and transportation to the leaves, by specific Na^+^ and K^+^ transporters, stomatal closure, ROS scavenging by induction of antioxidative enzymes (e.g., superoxide dismutase, SOD; catalase, CAT; ascorbate peroxidase, APX; etc.) and the induction of plant hormones (e.g., abscisic acid, ABA; auxin, cytokinin, CK; jasmonic acid, JA; salicylic acid, SA; strigolactones, SLs; etc.) (Munns and Tester, 2008; Deinlein et al., 2014; Evelin et al., 2019).

**FIGURE 2:**
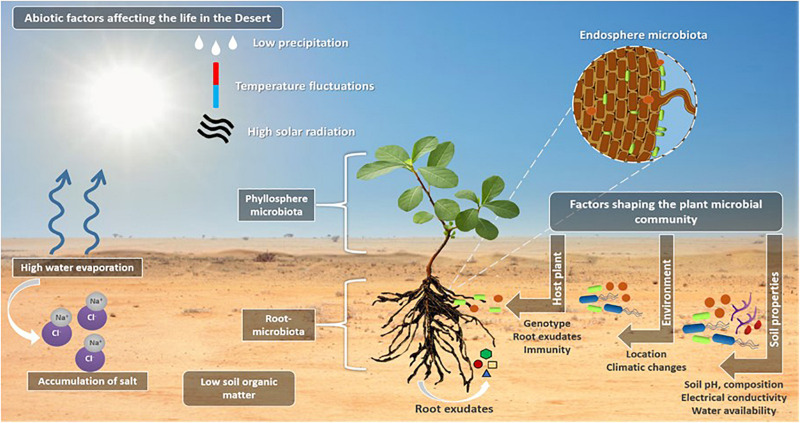
The life in the desert. The illustration highlights the abiotic factors affecting the life in the deserts, such as the low precipitation and high temperate fluctuations, high solar radiation which results in high water evaporation that result in salt ions accumulation in the soil causing soil salinity and affecting the soil composition. Plants in the deserts live in close association with the microorganisms that are inhabiting the plant aerial parts (phyllosphere microbiota), inside the plant root and in the zone around the root (root microbiota) where the root exudates are secreted to attract the bulk soil microbes. A number of factors including soil properties and composition, the water availability, the soil electrical conductivity and pH are responsible to shape the plant microbial communities and filter the selected microbes to improve fitness of plant and microbes. A second factor is the location and the associated climatic conditions, and finally, the host plant for the selection of the most beneficial associations, through means of plant defense mechanisms and immunity, root exudate secretions (this includes different quorum sensing mimicking compounds) and the host plant genotype.

In addition to the genetic and morphological adaptation to the desert environments, desert plants interact with microbial communities colonizing and inhabiting the surrounding soils (Vejan et al., 2016). For example, the rhizosheath represents a rich niche for microorganisms (e.g., nitrogen-fixing bacteria) and plant growth promoting bacteria (PGPB) (Othman et al., 2004; Ashraf et al., 2006; Hanna et al., 2013; Tahir et al., 2015). Microbes are also found in the aerial parts of plants (phyllosphere), inside the plant (endosphere), on the root surface (rhizoplane) and in the zone around the roots (rhizosphere and rhizosheath), where plant roots secrete a wide variety of compounds [e.g., sugars, amino acids, organic acids, proteins, mucilage, quorum sensing (QS) mimic compounds, etc.] in the form of root exudates in the rhizosphere, to attract microbial communities to this rich nutrient zone from the rest of the bulk soil (Haichar et al., 2014). The different factors that contribute to the recruitment, selection, enrichment, and dynamic interactions of plant-associated microbes are reviewed in details in Saad et al. (2020) and summarized in Figure 2.

## Bacterial Diversity and Factors Shaping the Bacterial Community in Desert Plants

In order to develop a sustainable agriculture, numerous research groups have focused toward desert plants and their associated microbial communities, living in extreme conditions such as drought, heat, and salinity. The introduction and application of culture-dependent and -independent methods have paved the way to exploring the microbial communities associated with plants (Lundberg et al., 2013). In recent years, massive parallel sequencing technologies enhanced the identification and quantification of microbial communities from various plant compartments (soil, rhizosphere, rhizosheath, endosphere, and phyllosphere), allowing insight beyond individual isolates (Lundberg et al., 2012, 2013; Lebeis, 2014; Eida et al., 2018; Acuña et al., 2019). Here, we will explore culture–independent amplicon sequencing studies that focus on the bacterial communities associated with desert plants from various regions in order to gain a better understanding of the bacterial communities colonizing different plant compartments and discuss some of the key factors that can shape and determine the bacterial community.

In an effort to inspect the factors influencing rhizosphere bacterial community, Na et al. (2018) investigated the rhizosphere bacterial community of the widely distributed perennial shrub *Caragana* in the arid and semi-arid areas of north China. The authors revealed the bacterial communities dominating the rhizosphere across three *Caragana* species belonged to Proteobacteria, Actinobacteria, Firmicutes, Bacteroidetes, Acidobacteria, Gemmatimonadetes, and Cyanobacteria, while on the genus level *Pseudomonas*, *Acinetobacter*, *Bacillus*, *Stenotrophomonas*, *Burkholderia*, *Paenibacillus*, *Sphingobacterium*, *Chitinophaga*, *Arthrobacter*, and *Chryseobacterium* were the most dominant genera. Furthermore, their results show that the soil properties such as relative humidity, pH, temperature, electric conductivity, and soil composition cooperatively shape the rhizosphere bacterial community structure, with emphasis on pH as a major factor that significantly influences the bacterial diversity and richness (Na et al., 2018). The data above, confirm that the geographical location and soil properties have a significant effect on the bacterial community, where similar bacterial rhizosphere communities are similar in closer geographical sites with similar soil type. In contrast, distant geographical sites with distinct soil properties harbor diverse bacterial communities. In another study in the Sonoran desert Finkel et al. (2012), show that also the phyllosphere bacterial community is under the influence of the geographical location and soil type. Furthermore, an interesting study by Marasco et al. (2018) supports this concept of the bacterial community being shaped by the desert soil effect over the imposed host plant genotype effect. The authors show that the microbial communities associated with the rhizosheath-root system of desert dune Spear grass (*Heteropogon contortus*) are stochastically assembled and host-independent (Marasco et al., 2018).

In addition to the geographical location and soil properties, the host plant genotype also plays an important role in shaping and selecting the bacterial community. In order to study the bacterial biodiversity and identify the driving factors, which determine and shape the community in the rhizosphere of desert plants, Osman et al. (2016) revealed the bacterial communities associated with the rhizosphere of four pioneer desert plants (the desert grass *Panicum turgidum* (Pt), and three dicotyl species that belong to the same (Zygophyllaceae) family: *Tribulus terrestris* (Tt), *Tribulus pentandum* (Tp), and *Zygophyllum simplex* (Zs), and in non-rhizosphere soil samples (Ss). The five samples were collected from the southern region (Jizan) in Saudi Arabia, from three different locations with different soil composition of the same desert. The results revealed a large diversity of bacterial communities found among the five samples (Pt, Tt, Tp, Zs, and Ss), where the most abundant members belonged to the Proteobacteria phylum in the rhizosphere of Tp, Tt and Zs, while the Pt sample had 79.1% bacterial members belonging to the Bacteroidetes phylum, with 68.1% of the OTUs are of the genus *Flavobacterium*. On the other hand, the soil sample (Ss) had the most abundant members belonging the Firmicutes phylum, with the highest abundance of members belonging to the genus *Bacillus*. These data suggest that, in spite of the extreme conditions, these pioneer desert plants harbored a large bacterial biodiversity in their rhizospheres. Moreover, the fact that three different plant species in three different sites with different soil properties (Tp, Tt, and Zs) had the most similar bacterial composition, suggests that abiotic conditions, such as geographical location, soil compositions, and soil properties, contribute to shape the plant rhizosphere bacterial community composition. At the same time, the Pt grass plant sample harbored the most distant bacterial composition, in comparison to the other three dicotyl species that belong to the same plant family, which suggests that host plant genotype plays another role in shaping the rhizosphere bacterial composition (Osman et al., 2016). Indeed, it was found that the soil type is the major key factor, followed by the host plant effect as secondary factor in determining the microbial community composition (Lundberg et al., 2012).

Another study that aims to reveal the bacterial community in pioneer desert plants from Saudi Arabia, and investigate the geographical and host plant effect in different plant compartments was done by Eida et al. (2018), where they examined the bacterial communities in the soil, rhizosphere and endosphere of four native desert plant species (*T. terrestris*, *Z. simplex*, *P. turgidum*, and *Euphorbia granulata*) from two different locations (Jizan and Al Wahbah Crater). The authors found that the bacterial communities in all samples (soil, rhizosphere, and endosphere) from both locations belonged to seven major bacterial phyla, with Actinobacteria and Proteobacteria dominating the rhizosphere and endosphere of all the plant samples. Interestingly, there was a gradual increase of Actinobacteria abundance from the soil to the rhizosphere to the endosphere. In addition, some of the bacterial genera that have been isolated belonged to *Rhizobium*, *Bacillus*, *Pseudomonas*, *Kocuria*, and *Microbacterium*. These results show that the bacterial communities were significantly different between the soil of Al Wahbah and Jizan samples, correlating with the major differences in soil composition between the two locations. However, despite of the geographical location, the endosphere samples of the same plant species cluster together, indicating a host plant genotype effect (Eida et al., 2018).

In the semi-arid regions of Mexico, the Cacti microbiome was analyzed in six plant compartments: the rhizosphere, the phyllosphere of the stem surface, stem and root endosphere, the surrounding bulk and root-zone soils, in two Cacti species: *Myrtillocactus geometrizans* and *Opuntia robusta* at two different geographical locations with similar semi-arid climate and soil characteristics (Fonseca-García et al., 2016). The authors revealed that the bacterial community belonged to Proteobacteria, Actinobacteria, Bacteroidetes, Acidobacteria, and Firmicutes, with a relative abundance of more than 85% in the rhizosphere, root endosphere, stem endosphere, and the phyllosphere in both cacti species. In addition, the *Bacillus* genus dominated the rhizosphere, while members of the Gammaproteobacteria and cyanobacteria dominated the phyllosphere, Actinobacteria and Alphaproteobacteria in the root endosphere, and another *Bacillus* and Sphingobacteria taxa that belongs to the Bacteroidetes dominated the stem endosphere of both cacti species. These results show that the bulk and root-zone soils had similar bacterial composition. However, the root, stem endosphere, and phyllosphere differed clearly from the soil bacterial communities, and the bacterial composition within each of these compartments were distinct from other plant compartments and similar among the two cacti species, which shows that the host plant has a major influence on the bacterial community. Finally, the reason behind the abiotic factors such as season, geographical location and soil properties not effecting the bacterial community in this case, could be due to the fact that both sampling sites had similar ecological factors of climate and soil composition, leaving the host plant effect as the main driver of selecting and shaping the cacti bacterial community (Fonseca-García et al., 2016).

We can conclude here, that desert plants harbor diverse bacterial communities mainly dominated by two major bacterial phyla the Proteobacteria and Actinobacteria, and in order to better understand the enrichment of these specific bacterial phyla with the desert plants, we will discuss some of their major functions and characteristics. First, Proteobacteria: this phyla and more specifically the alpha-proteobacteria class, show high adaptability to a wide range of environmental lifestyles, some of the most common genera found within this class are specific plant symbionts with nitrogen fixing capabilities such as *Rhizobium, Mesorhizobium, Sinorhizobuim*, and *Azorhizobium* (Pini et al., 2011). Second, Actinobacteria: bacterial members belonging to this phyla, show strong versatility of growing under extreme selective conditions such as salinity, low and high pH, low water availability, extreme temperatures, radiation, and pressure, they embrace a complete spectrum of members living in extreme ecosystems (e.g., alkaliphilic, acidotolerant, thermotolerant, and halotolerant strains) (Mohammadipanah and Wink, 2016). On the genus level, Actinobacteria harbor some of plant growth promoting conferring genera such as *Streptomyces*, *Saccharomonospora*, and *Nocardioides* (Yandigeri et al., 2012; Nafis et al., 2019). Finally, it is clear that desert habitats exert selective pressures on their soil and plant associated bacterial communities to choose the best-adapted phyla to help them thrive in such extreme conditions.

Furthermore, it is clear that the bacterial communities associated with desert plants are most certainly under the influence of many biotic and abiotic factors (e.g., soil composition and properties, temperature, pH, seasons, climate change, geographical location, host plant, etc.), which can shape the taxonomic and functional groups of the community in different plant compartments (e.g., rhizosphere, rhizosheath, endosphere, and phyllosphere). In addition, one of the key factors is the soil type and properties, associated with the plants geographical location, hence, similar geographical locations have similar soil, rhizosphere and phyllosphere bacterial community, while plants in distant geographical locations with distinct soil properties have distinct soil bacterial community and vary in their associated bacterial communities. It is also clear that the plant host-genotype have an effect in selecting the endosphere bacterial community. However, this factor is considered to be of a secondary effect. Therefore, the geographical location, season, soil type and properties determine the bulk soil, phyllosphere and rhizosphere bacteria, and then the plant selects the best bacterial partners to ensure the best fitness and survival for both plant and its associated bacterial community. Finally, it is important to note that this is not only true for bacterial communities in desert plants, but applies to the whole microbial community associated with desert plants such as fungal and archaeal community. For example, it seems that the fungal community is mainly influenced by the host plant genotype, soil type and biogeography (Coleman-Derr et al., 2016; Fonseca-García et al., 2016; Suleiman et al., 2019).

In brief, there is a huge bacterial diversity in desert plants selected specifically to fit the plant’s needs and among those are beneficial microbes, which have been increasingly cultured and tested for promoting plant growth and health.

## Application of Desert PGPR for Sustainable Agriculture

Desert habitats contain a large diversity of microbial communities functioning as plant mutualists that can directly interact with their host plant, providing fundamental processes in exchange for niche and nutrients, where both partners are benefiting from this symbiotic relationship to improve the fitness of both organisms (Vacheron et al., 2013). Some of the major plant mutualists are nitrogen-fixing bacteria, mycorrhizal fungi, and plant growth promoting rhizobacteria (PGPR) (Mus et al., 2016).

Plant growth promoting rhizobacteria are the group of microbes that proliferate in the host plant rhizosphere and stimulate plant growth due to specific traits they possess, they can enhance plant growth by direct and indirect mechanisms in order to enhance plant physiological processes and pathogen resistance (Vejan et al., 2016). The direct mechanisms include nutrient mobilization such as nitrogen fixation, phosphate and potassium solubilization, iron sequestration in the soil and the regulation of phytohormones and growth regulators such as indole-3-acetic acid (IAA), and aminocyclopropane-1-carboxylic acid (ACC) deaminase (Bhattacharyya and Jha, 2012). On the other hand, the indirect mechanisms include protecting plants against plant pathogens by inhibiting or controlling them through the production of antagonistic compounds, such as hydrolytic enzymes, production of volatile organic compounds (VOCs), QS signaling molecules and antibiotics (Vejan et al., 2016; Altaf et al., 2017; Gouda et al., 2018).

For social communication and environmental sensing both beneficial and pathogenic bacteria use QS. QS is a regulatory mechanism that works on the gene level, where bacteria produce a wide range of small singling molecules known as auto-inducers (AI) (Khan et al., 2020). Bacteria release AIs into the environment to assess their surroundings, and help them adapt their gene expression in response to the given conditions to optimize their population number and fitness. Moreover, one of the well-studied AI produced by Gram-negative bacteria is the *N*-acyl-homoserine lactone (AHL), while cyclic peptides are mostly produced by Gram-positive bacteria (Hartmann et al., 2014). In addition, several bacterial physiological behaviors such as symbiosis, virulence, antibiotic production, conjugation, competence, sporulation, and biofilm formation are controlled by QS (Rutherford and Bassler, 2012).

It is noteworthy that some PGPR use QS for the communication with members of the same and other bacterial species, but also with higher organisms, i.e., plants. Interestingly, for plant–microbe communication, it is evident now that the QS communication is essential for the microbial interactions at the rhizosphere (Altaf et al., 2017). During evolution, plants have learned to respond to QS via several means such as sensing and responding to the bacterial QS signals and producing AHL-mimic substances that can influence QS in the plant-associated bacterial community. Moreover, several studies have revealed that the rhizosphere harbors more AHL producing bacteria than the rest of the bulk soil, and that these AHLs can work as inter-kingdom signaling molecules (Elasri et al., 2001; Altaf et al., 2017). The AHL producing bacteria, e.g., *Burkholderia*, *Pseudomonas*, and *Sinorhizobium* can affect plant gene regulation, stimulate plant growth and development and initiate systemic resistance to pathogens. Furthermore, QS helps in selecting and shaping the bacterial partners with specific PGPR and biocontrol functions thereby benefiting plants to achieve optimum fitness (Hartmann et al., 2014; Altaf et al., 2017; Zaytseva et al., 2019). Such PGPR can be found enriched in the rhizospheres of desert plants such as Agave and cacti (Flores-Núñez et al., 2020).

Desert PGPR are evolutionary well adapted to extreme environmental conditions such as heat and high salinity, exploiting stress response genes to promote plant growth and enhance soil fertility more than microbes found in non-arid soils (Paul and Lade, 2014; Makhalanyane et al., 2015; Eida et al., 2018; Bokhari et al., 2019; Eida et al., 2019). They grow with desert plants in intimate mutualistic interactions, helping them to thrive in the extreme conditions, providing fundamental nutrients and enhancing plant tolerance to abiotic and biotic stresses. Thus, desert plants display a perfect niche for studying the bacterial diversity as well as isolating their associated PGPR. In order to exploit them for their magnificent capabilities to enhance plant growth and resistance in arid, semi- and hyper-arid environments for obtaining more fertile soil, higher crop yields, increased agriculture sustainability to help improving the desert framing (Marasco et al., 2012; Cherif et al., 2015; Daur et al., 2018; de Zélicourt et al., 2018).

Taking into account the importance of desert plant associated microbes; we will review below the potential of desert microbes for improving sustainability of agriculture in in the arid, semi-arid and hyper-arid regions of the world, with the focus on the PGPB applications in the Arabian Peninsula Deserts.

## The South American Deserts

In a recent study in the Andean Altiplano of the Atacama, the rhizosphere of the native shrub *Parastrephia quadrangularis* was characterized and selected for ACC deaminase producing rhizobacteria (Acuña et al., 2019). The authors found 10 representative strains that belonged to the Enterobacteriaceae family and displayed ACC deaminase activity, high tolerance to salt and produced auxin and siderophores. Furthermore, in order to test their PGPR capacities under salt stress, two ACC deaminase producing *Klebsiella* strains were selected for the inoculation with wheat (*Triticum aestivum* L.) seedlings. The results showed a significant increase in the plant biomass (45–62%), as well as a significant SOD response in the roots. The increased SOD response under salt stress, reflects the antioxidant activity against salt stress-induced ROS production caused by the salt stress. This study confirms that indeed, PGPR associated with native plants growing in extreme environments such as the Atacama desert, can be a rich source for novel PGPR to help plants tolerate salt stress (Acuña et al., 2019).

To study the impact of several PGPR isolated from the Chilean deserts, and their potential role in assisting desert plants to face drought and nutrient deficiency stresses, Inostroza et al. (2017) investigated the effect of PGPR consortia that have been previously isolated from arid ecosystems and agro-ecosystem, on wheat plants under stressed conditions. The selected bacterial consortia used in this study are as follows: Consortium (C1) from an arid ecosystem (two *Bacillus* strains and one *Serratia* strain), isolated from the rhizosphere of *Atriplex* species from the Atacama and Aconcagua Valley, and Consortium (C2) from an agro ecosystem (two *Enterobacter* strains and one *Bacillus* strains). In addition, the wheat plants were grown in two experimental setups; in pots under growth chamber conditions with water shortage and different soil composition (stressed condition), and in pots in an open greenhouse under (natural conditions) for 30 days. The results revealed that, under the normal conditions, both consortia had positive PGP effects on the wheat plants. However, under water shortage conditions and poor phosphorus soil composition similar to desert conditions, Consortium C1 from the arid ecosystem exhibited higher PGP impact compared to Consortium C2 (Inostroza et al., 2017). This interesting finding, demonstrates that PGPR isolated from agricultural ecosystem exhibit PGP effects by increasing wheat biomass in natural, but not in stressed conditions, which suggest that these isolates are more adapted with PGPR traits that best fits their native host plant environments. Likewise, PGPR isolated from desert plants exhibit PGP effects in both natural and stressed conditions, which suggests that indeed, they are more adapted to abiotic stresses such as drought and nutrient deficient soils that resemble desert conditions. Therefore, desert plants display the prefect source for isolating PGPR equipped with traits that can survive in extreme conditions.

## The North American Deserts

Soil degradation is one of the major abiotic threats in the Sonoran Desert, due to wind and water erosion that result in the removal of the BSC, which contains the majority of the microbial life crucial for plant–microbe association (Jeong and Dorn, 2019). It is challenging to restore the degraded soil areas by simple traditional means such as implanting seeds and supplying water. Therefore, scientists have focused more on isolating native PGPR, reintroducing them as bio-inoculants to enhance soil fertility and plant growth in the affected areas. In efforts to pursue this goal, Bashan et al. (2012) conducted a field study in the Sonoran Desert, on native legume trees *Mesquite amargo*, *Prosopis articulata*, *Parkinsonia microphylla* (yellow paloverde), and *Parkinsonia florida* (blue paloverde), where they inoculated the trees with two native PGPR strains (*Azospirillum brasilense* and *Bacillus pumilus*) and AM fungi. Their results showed that, the mesquite and yellow paloverde trees had the most significant responses to the PGPR’s and AM fungi in terms of height, diameter of the main stem and number of branches after 3 months. In addition, they showed that the plant growth parameters varied according to inoculation and depended mainly on the tree species. This study demonstrates that indeed, restoration and revegetation of degraded soils is possible through novel means of isolating native PGPR and beneficial fungi associated with plants surviving in degraded soil areas, and then reintroducing them as beneficial inoculants that can have various positive effects on the soil and the plants (Bashan et al., 2012).

In another example from the Sonoran desert (Galaviz Bustamante et al., 2018), isolated an endophytic PGPR strain (*B. pumilus*) from the rhizoplane of a cactus surviving in a degraded soil condition, and then reintroduced *B. pumilus* as a inoculant with a mesquite tree (*P. articulata*), which is found in the same degraded soil and under the same desert climatic conditions. Their data demonstrated that the inoculation of *B. pumilus* has significantly enhanced the mesquite tree roots biomass and changed the bacterial community structure in both the rhizosphere and non-rhizosphere soil. In addition, in the rhizosphere of the inoculated plants, there was a significant increase in the relative abundance of Proteobacteria and Acidobacteria phyla, with a decrease in Actinobacteria. Interestingly, the inoculation of *B. pumilus* increased the abundance of Rhizobia strains in the mesquite rhizosphere, which is assumed to be due to the reactivation of rhizobia; due to a higher carbon content after the reintroduction of the PGPR and restoration of the soil fertility (Galaviz Bustamante et al., 2018).

In the Mojave Desert, analysis of the *Larrea tridentata* rhizobacterial community revealed the most abundant species belonged to Proteobacteria, Bacteroidetes and Firmicutes phyla (Jorquera et al., 2012). Additionally, when three representative isolates from *L. tridentata* were tested for their PGPR activities, the strains encoded for ACC deaminase and phytases, which indicates their PGPR capability to utilize phosphate by possessing phytase enzymes, which can mineralize phytate, the predominant form of organic phosphate in the soil. In addition, to confirm the PGPR effect of these isolates, the strains were further inoculated with cucumber plants, where they showed three fold higher root and shoot biomass than control plants. This study supports the hypothesis that desert plants harbor a large diversity of PGPR that can contribute to an increase of plant fitness, by several means such as phosphate solubilization (Jorquera et al., 2012).

In the Chihuahuan Desert, a study was conducted on the native halophilic perennial grass *Distichlis spicata*’s roots, with the aim of isolating and characterizing its potential beneficial rhizobacteria, and further testing them on Arabidopsis and crop plants under salt stress (Palacio-Rodríguez et al., 2017). The authors show two representative PGPR strains (*Bacillus* sp.) and (*Pseudomonas lini*) exhibited the strongest effect on enhancing the shoot and root biomass in normal and salt stress conditions, both PGPR strains exhibited halophilic properties, IAA and siderophores production, as well as expression of the *acdS* gene (ACC deaminase) by *P. lini.* Additionally, the inoculation of the two strains with *Arabidopsis thaliana*, cucumber and watermelon under normal and salt stress conditions, showed higher plant biomass and increased lateral root number. In this study, the isolation and characterization of the two halophilic PGPR strains from the desert plant *D. spicata*, reveals a great potential approach of isolating novel halophilic PGPR strains that can strive in extreme saline conditions, and further apply them to improve crops growing in salt-affected regions (Palacio-Rodríguez et al., 2017).

## The Great Indian Desert (Thar Desert)

In an attempt to improve and sustain the production of horticultural crops growing in arid areas, such as the Thar Desert, Aseri et al. (2008) investigated the effect of several nitrogen fixing PGPR strains (individually), and in a dual combination of one PGPR strain (*Azotobacter chroococcum*) with the beneficial AM fungi (*Glomus mosseae*) on the production of the commercial fruit, Pomegranate (*Punica granatum* L.) in the span of 5 years field study. Their results revealed that the individual inoculation with the PGPR strains significantly enhanced the plants height, plant canopy and fruit yield, in addition to higher chlorophyll content and nutrient uptake. Interestingly, the dual inoculation of the PGPR strain with the AM fungi displayed the best results, with the maximum increase in all of these parameters. These data suggest that the use of PGPR as bio-fertilizers, that were isolated from the rhizosphere of plants growing in arid areas such as the Thar Desert, can be effectively applicable to improve horticultural crop plants in harsh desert areas (Aseri et al., 2008).

In the semi-arid Rajasthan region of the Thar Desert, a rhizobacterial strain was isolated from which mung bean (*Vigna radiate*) and identified as *Bacillus* sp. displayed beneficial PGPR traits such as phosphate solubilization, antifungal activity, ACC deaminase activity and ammonia and IAA production (Minaxi et al., 2012). Furthermore, this PGPR was further inoculated with the crop cowpea under semi-arid conditions in laboratory and field conditions, and displayed positive enhancement of plant growth and nutrient uptake in the cowpea plants. In addition, coated seeds with the *Bacillus* isolate showed a significant increase in seed germination, shoot and root length, fresh and dry weight and increased leaf area. In the field, the number of pods, seeds and grains were significantly enhanced compared to control non-inoculated plants. These data show that PGPR isolated from plants growing in semi-arid areas can have remarkable PGPR traits, which can positively enhance the growth and production of important food crops such as cowpea plants, and other fundamental crops in the semi-arid regions across Asia (Minaxi et al., 2012).

Another study in the Rajasthan region of the Thar Desert, on the widely distributed shrub plant *Capparis decidua* (Forsk.) Edgew, which shows great capacity to grow extreme hot and dry conditions in the arid and semi-arid regions of the Thar, and display alkalinity and salinity tolerance, as well as unique capacity to withstand the drought and heat stress (Singh and Jha, 2016). Moreover, a strain identified as *Serratia marcescens* was isolated from the rhizosphere of this plant, exhibited several PGP traits such as: ACC deaminase activity, salt tolerance, phosphate solubilization, IAA production, siderophores production and the ability to fix ammonia and nitrogen. In addition, the inoculation of *S. marcescens* with wheat plants under salt stress, significantly enhanced the plant growth, and increased the osmo-protectant levels of proline, malondialdehyde, total soluble sugar, and IAA and altered the antioxidant activities of several antioxidant enzymes (e.g., SOD, CAT, and peroxidase). Additionally, PGPR inoculation with wheat plants upon fungal infection, has significantly reduced the fungal disease severity and conferred the plants with induced systemic resistance against the disease. Finally, these results reveal a potential bio-fertilizer with great PGPR activity against both biotic and abiotic stresses, which was originally isolated from a desert plant thriving and growing in extreme hot, saline and dry desert conditions (Singh and Jha, 2016).

## The North West Deserts of China

The perennial plant *Alhagi sparsifolia* is considered to be one of the most dominant desert plant species in the extreme dry and saline region of the Taklamakan Desert, in the north-west regions of the Gobi Desert in China (Chen et al., 2016). This plant has deep roots that are highly developed to enhance its capacity to withstand drought and poor soil conditions. In efforts to identify and characterize it’s associated endophytic bacterial communities, Chen et al. (2017) isolated bacteria from different parts of the plant (leaves, stems, and roots), and subjected the isolates to *in vitro* tests to characterize their PGPR capacities. In addition, the tests revealed a novel bacterial isolate as a member of the genus *Pantoea*, which showed significant drought tolerance capacity, as well as osmotic, salt and heat tolerance, along with several PGP traits, such as phosphate solubilization, production of IAA, siderophores, exopolysaccharides (EPS), and proteases. In addition, inoculation of this PGPB strain with wheat plants under drought stress showed that this endophytic PGPB successfully promoted wheat plant growth under drought stress, and increased the soluble sugars content, chlorophyll content in the leaves accompanied with decreased proline levels, which indicate drought stress tolerance in the wheat plants. These data display the great effect of this endophytic PGPB in conferring the crop plant with drought stress tolerance. In addition, this PGPB, which is originally isolated from a highly adapted desert plant to drought stress confirms that plants surviving in extreme conditions of drought stress, can give a wide variety of novel PGPB that can be utilized in the future as bio-fertilizer, to help crop plants grow and face extreme dry desert conditions.

The desert plant *Salicornia europaea L.* (Chenopodiaceae) is considered to be one of the most dominant pioneer salt accumulating halophytes in the arid saline region of the Gurbantunggut Desert in China (Zhao et al., 2016). With the goal of isolating and characterizing the endophytic communities of *S. europaea*, Zhao et al. (2016) isolated the bacterial communities from different plant compartments (root, stem, and assimilation twigs). Their results revealed 105 bacterial isolates, where 32 of which were selected for ACC deaminase and IAA production and phosphate solubilization. In addition, for further screening for salt tolerance promoting bacteria, *S. europaea* was inoculated with the bacterial isolates under salt stress conditions. Their results revealed five bacterial isolates with the highest plant growth and salt tolerance beneficial impact. The bacterial isolates were later identified as *B. endophyticus*, *B. tequilensis*, *Planococcus rifietoensis*, *Variovorax paradoxus*, and *Arthrobacter agilis*. These results show that these endophytic salt tolerance promoting PGPB associated with the halophytic plant, are well adapted to high salt concentrations and assist halophytic plants to tolerate abiotic stresses such as soil salinity found in the deserts (Zhao et al., 2016).

## The South African Deserts

In the arid parts of South Africa, more specifically the eastern parts of Namibia, an indigenous (non-nodulating legume) plant known as marama bean (*Tylosema esculentum*), has strong capacity to thrive in nitrogen deficient habitats and to synthesize high protein contents in their seeds (Chimwamurombe et al., 2016). The group of Chimwamurombe et al. (2016) demonstrated the presence of seed transmittable bacterial endophytes with growth promoting effects, and their potential role for resilience to the harsh desert conditions. Interestingly, a total of 123 bacterial isolates were isolated and identified, 73 of which were putative endophytes that belong to bacterial species from 14 genera including Proteobacteria (*Rhizobium*, *Massilia*, *Caulobacter Pseudorhodoferax*, *Pantoea*, *Sphingomonas*, *Burkholderia*, *Kosakonia*, and *Methylobacterium*), Firmicutes (*Bacillus*), Actinobacteria (*Microbacterium* and *Curtobacterium*), and Bacteroidetes (*Mucilaginibacter* and *Chitinophaga*). Further screening for PGPB isolates, revealed 29 isolates that exhibited PGP activities such as the production of IAA, ACC deaminase, siderophores, proteases, and phosphate solubilization and most importantly nitrogen fixation capacities. This study reports the association of the high protein marama bean seeds with a large number of endophytic beneficial bacteria, and displays the adaptive strategy of these plants to associate their seeds with nitrogen fixing PGPB, in order to assist their growth and survival in nitrogen deficient arid environment. The isolation of novel endophytic PGPB that are adapted to such poor soil conditions can be a potential strategy to use them as inoculants for poor soils in the arid regions (Chimwamurombe et al., 2016).

The Kalahari truffle (*Kalaharituber pfeilii*), is an edible mycorrhizal fungus that is found in the Kalahari Desert in South Africa (Adeleke and Dames, 2014). This truffle is unable to complete its life cycle without a host plant and in order to understand this association, Adeleke and Dames (2014) looked for other positive potential partners that can facilitate this association such as bacteria; these bacteria are known as mycorhization helper bacteria (MHB), which can stimulate the fungal growth and display PGPR activities. In addition, the results revealed 17 bacterial isolates from Kalahari truffle that belonged to Proteobacteria, Firmicutes and Actinobacteria phyla. Interestingly, some of these isolated displayed PGP traits such as IAA production and phosphate solubilization, as well as stimulation of the mycelial growth *in vitro*, which indicate potential MHB that display PGP traits, stimulate and assist the fungal growth in desert conditions (Adeleke and Dames, 2014).

## The North African Deserts

Plant growth promoting rhizobacteria in the North African deserts such as Sahara Desert are under extensive research, where several studies identified potential PGPB strains from different sources such as soil, rhizosphere, and endosphere, which can help different plant species against abiotic and biotic stresses imposed by these extreme conditions. In the Tunisian Sahara Desert, oases represent natural agro-systems where desert plants such as date palm trees, can grow in a more fertile soil condition than the surrounding desert soils (Cherif et al., 2015). In an attempt to study and characterize the bacterial community associated with the date palm (*Phoenix dactylifera* L.) roots, Cherif et al. (2015) isolated the endophytic bacterial community, where the majority of the bacterial isolates belonged to Proteobacteria, Actinobacteria, Firmicutes, and Bacteroidetes phyla. In addition, screening the isolates for their PGPB traits revealed several PGP traits such as salt tolerance, drought, and variable temperatures resistance. Moreover, the most abundant endophytic bacterial isolates belonged to the *Pseudomonas* genus; hence, two selected *Pseudomonas* strains with the greatest PGP capacities were inoculated with the date palm to assist their PGP activity under drought conditions. The results show that the strains successfully increased date palm biomass under drought conditions, suggesting that date palms of desert farming oases can shape their endophytic microbial communities in a way to promote their growth under drought conditions (Cherif et al., 2015).

With the purpose of isolating and characterizing potential bacterial strains associated with desert plants, surviving and adapted to the Algerian Sahara Desert, Goudjal et al. (2013) isolated the endophytic bacteria from five random desert plants that are well adapted to the Sahara conditions. In addition, the majority of the isolated strains belonged to the Actinobacteria phylum, and more specifically (*Streptomyces*) genus, hence, the authors further chose a *Streptomyces* sp. PT2 strain that had the highest production of IAA. The treatment of tomato cv. Marmande seeds with the IAA-producing supernatant culture of the PT2 isolate showed maximal promotion of seed germination and root elongation. These results indicate that this endophytic PGPB has the ability to produce plant growth regulators such as auxin, which can contribute to protect the desert plants and stimulate their growth in extreme desert environments (Goudjal et al., 2013). It is clear that PGPB isolates found associated with the Saharan desert plants, act as bio-fertilizers that help the plants against abiotic stress conditions such drought, salt, and heat stress. However, there is also vast majority of PGPB that are isolated from the Saharan soils, and plants which can act as bio-control agents, and help against biotic stresses such as plant pathogens (Muzammil et al., 2014).

A potential biocontrol *Streptomyces* strain was isolated from the roots of the Saharan native plant *P. turgidum* (Zamoum et al., 2017), this *Streptomyces* strain was identified as *S. rochei* strain PTL2 and showed a number of PGP traits including the production of hydrogen cyanide (HCN), siderophores, ACC deaminase, and phosphate solubilization. Furthermore, this PLT2 strain showed strong antagonistic activity against the soil-borne pathogenic fungus *Rhizoctonia solani*, revealing the potential use of this desert PGPR as a biocontrol agent, which could help various crop plants against plant pathogens (Zamoum et al., 2017). In addition, *Nocardiopsis dassonvillei* MB22 with a strong biocontrol activity was isolated from the Saharan soils and showed several PGP traits including the production of IAA, HCN, and phosphate solubilization (Allali et al., 2019). Wheat (*Triticum* spp.) seedlings inoculated with the pathogenic fungus *Bipolaris sorokiniana* showed a significant reduction of infection from 90.8 to 27.7%, including an increase in dry weight, root and shoot length, which indicates the dual function and potential of this desert PGPB as a bio-fertilizer and biocontrol agent (Allali et al., 2019).

## Showcase: the Arabian Peninsula Deserts

Several initiatives were launched to discover the possibility of using desert microbes from the Arabian Peninsula Deserts, including the DARWIN21 project^1^. The project was started with the idea to explore the diversity of the microbial communities of desert plants and utilize them to improve agricultural sustainability on arid land. Within the DARWIN21 framework both culture-dependent and culture-independent methods were employed to identify and characterize bacterial communities of desert microbes from different desert plant species (Osman et al., 2016; Bang et al., 2018; Eida et al., 2018). Hitherto, the DARWIN21 project has generated over 2,500 cultivable endophytic bacterial strains from various deserts in the Middle East (Bang et al., 2018). A number of DARWIN21 bacterial strains were genome sequenced (Andrés-Barrao et al., 2017; Eida et al., 2020a, b,c) providing a platform for genome mining and comparative genomics studies to identify the genes responsible for conferring stress tolerance and survival of desert and other plants as well explaining the PGPR activities. The potential of the plant growth promoting activities including nutrient acquisitions as well as helping the plant to grow under abiotic stress, e.g., salt and drought and fighting the plant pathogens were conducted (Andrés-Barrao et al., 2017; Bokhari et al., 2019; Eida et al., 2020c). Moreover, the mechanisms employed by a number of those strains were highlighted the involvement of the ethylene regulation and ions homeostasis to help plant to tolerate the salt stress (de Zélicourt et al., 2018; Eida et al., 2019).

Other research groups were actively working on studying the microbial population and translation of the knowledge to agriculture as bio-fertilizer, bio-control, and industrial application as well as in human health (e.g., new drugs and new antibiotics). For instance, the medical plant *Plectranthus tenuiflorus* is used to treat a wide variety of diseases such as skin, digestive, respiratory diseases as well as ear inflammations by the locals in Saudi Arabia, likewise this plant is used in Asia to treat sore throat (Rahman et al., 2004). In addition, the medical properties of this plant, have made it an interesting research subject for investigating its endophytic bacteria that might have PGP traits against bacterial human pathogens, hence, El-Deeb et al. (2013) isolated the endophytic bacteria from root, stem and leaves of *P. tenuiflorus*, where eight isolates were identified as *Bacillus* spp., *B. megaterium*, *B. pumilus*, *B. licheniformis*, *Micrococcus luteus*, *Paenibacillus* spp., *Pseudomonas* spp., and *Acinetobacter calcoaceticus*. In addition, most of these isolates exhibited extracellular enzymatic activity, and one *Bacillus* strain showed the highest production of extracellular enzymes including amylase, esterase, lipase, protease, pectinase, xylanase, and cellulose. Interestingly, this strain exhibited positive antimicrobial activity against several human pathogenic microorganisms such as: *Staphylococcus aureus*, *Escherichia coli*, *Klebsiella pneumoniae*, *Streptococcus agalactiae*, *Proteus mirabilis*, and *Candida albicans*. These results demonstrate the possibility of using desert PGPR as a source for antimicrobial and secondary metabolite production against several human pathogens (El-Deeb et al., 2013).

From the rhizosphere of pioneer plant species collected from different locations in North Jeddah, Saudi Arabia (Elazzazy et al., 2012), several isolates were identified as *B. subtilis, B. amyloliquefaciens*, and *P. aeruginosa*, and exhibited strong antagonistic activity against the fungal pathogen *Pythium aphanidermatum in vitro*. Three isolates showed plant growth promoting effects on cucumber (*Cucumis sativus* L. cv. Marketmore) by increasing plant height, stem length, fresh, and dry weight. Additionally, cucumber seeds coated with *B. amyloliquefaciens* and *P. aeruginosa* resulted in significant reduction of the damping-off disease of cucumber seedlings caused by *P. aphanidermatum*. This study demonstrates the potential of some PGPR to have a dual function as biocontrol agent and a bio-fertilizer that promotes crops yields (Elazzazy et al., 2012).

In the center of the Arabian Peninsula in Al-Qassim region, the antagonistic activity of a potential biocontrol endomopathogenic (*Cladosporium chlorocephalum*) fungus against sweet potato white fly (*Bemisia tabaci*) in cabbage was reported by Aldeghairi et al. (2013). The group showed that under laboratory conditions, when *B. tabaci* nymphs and eggs were infected with *C. chlorocephalum*, the nymphs showed higher sensitivity to the beneficial fungus than the eggs. This finding reports a potential biocontrol fungus isolated from a desert plant, which could be used as an alternative solution of chemical pesticides to fight the potato white fly disease.

In the province Khulays in the Makkah district of western Saudi Arabia, several bacterial isolates were obtained from the rhizosphere and rhizoplane samples of different desert plants by Almaghrabi et al. (2014). The bacterial isolates belonged to nine genera with twelve taxa including (*P. putida*, *P. fluorescens*, *P. areuginosa*, *Serratia marcences*, *Xanthomonas* sp., *B. cereus*, *Microccoucs* sp., *B. subtilis*, *B. megaterium*, *B. amyloliquefaciencs*, *Pseudomonas* spp., and *Staphylococcus* spp.). Three selected isolates (*S. marcences*, *P. putida*, and *P. fluorescens*) showed the highest IAA production and had a significant impact on tomato plants growth, showing increased seed germination, plant height, shoot dry weight, and root elongation (Almaghrabi et al., 2014).

In Hada Al Sham near Jeddah, western Saudi Arabia, El Sayed et al. (2015) isolated from rhizosphere of wheat plants, a nitrogen fixer and IAA producing strain identified as *Azospirillum brasilense* (HM1). Inoculation of the soil with the HM1 strain had a positive PGPR effect on wheat plant root length, fresh and dry weight of roots and shoots, as well as increased contents in protein, chlorophyll, phosphorous, nitrogen, and IAA. These results emphasize the potential of *Azospirillum* as a bio-fertilizer to help plant growth in nitrogen-deficient soils (El Sayed et al., 2015). In another study at Hada Al Sham, Daur et al. (2018) have isolated several root-adhering rhizobacteria from the desert plants (*Setaria viridis*, *Cenchrus ciliaris*, *P. antidotale*, *Amaranthus viridis*, and *Dichanthium annulatum*). The bacterial strains were identified as *Bacillus*, *Actinobacter*, and *Enterobacter*, the biochemical assays confirmed that all the isolates have PGP traits such as IAA, ACC deaminase, siderophores, phosphatase production, and solubilization. The isolates were assayed for their PGPR activities to improve the alfalfa (*Medicago sativa L.*) growth, nutrient uptake and yield. Inoculation of alfalfa plants with any of the isolated strains improved plant water content, chlorophyll, carotenoid, nitrogen, phosphorus, and potassium content as well as other plant growth parameters such as plant height, leaf to stem ratio, fresh and dry weight. This study demonstrates the potential of using the desert rhizobacterial strains to develop novel bio-fertilizers for the improvement of crop production in hyper arid regions such as Saudi Arabia (Daur et al., 2018).

In the Al Jouf region in North Saudi, a PGPB Actinobacteria isolate (Ac5) was obtained from the rhizosphere of the drought resistant desert plant *P. turgidum* by Selim et al. (2019). This Ac5 isolate could produce flavonoids, phytohormones and siderophores, and the inoculation of Ac5 with maize plants have significantly reduced the detrimental effects of drought stress. Ac5 treatment significantly improved plant growth, reduced ROS accumulation and lipid peroxidation and plants showed higher levels of antioxidants such as ascorbate, glutathione, tocopherols, flavonoids, and phenolic acids. In addition, the Ac5 inoculated plants also accumulated compatible solutes including soluble sugars, sucrose, proline, glycine betaine, and arginine. This study demonstrates the comprehensive effect of Actinobacteria as PGPRs to combat drought stress, and highlights the fundamental role of rhizobacterial communities in drought resistant desert plants (Selim et al., 2019).

At the coast of the Red Sea near city of Jizan, Saudi Arabia, several plant species were collected by Eida et al. (2019). The collected desert plants included the annual halophytes *T. terrestris and Z. simplex*, the salt tolerant perennial tussock-grass *P. turgidum* and the annual euphorbium *E. granulate* and further isolation of the endophytic bacteria was conducted to characterize their PGP properties *in vitro*. Additionally, six selected bacterial isolates belonging to Actinobacteria (*Cellulomonas* sp. Jz18, *Arthrobacter* sp. JZ12, and *Microbacterium* JZ37), Proteobacteria (*Pantoea stewartii* JZ2 and *P. stewartii* JZ29), and Firmicutes (*Bacillus* sp. JZ34) were inoculated with *A. thaliana* to assist plant growth and salt tolerance activity. Five of these isolates conferred significant increase in plant shoot and root weight under salt stress conditions, salinity stress also resulted in tissue specific transcriptional changes in the ion transporters and reduced Na^+^/K^+^ shoot ratio (Eida et al., 2019).

Several proof of concept and translational studies were done on an endophytic bacterial strain (*Enterobacter* sp. SA187), isolated from the desert legume plant *Indigofera argentea*, have showed different PGPR traits including indole and siderophore production as well as survival under high salt and low water content (Andrés-Barrao et al., 2017). *In planta*, SA187 significantly increased salt tolerance of *Arabidopsis thaliana* and different crop plants under greenhouse conditions. Under desert farming conditions, SA187 increased the biomass of alfalfa (*M. sativa*) using saline water irrigation (de Zélicourt et al., 2018). Moreover, the use of SA187 to help plants to tolerate abiotic stress and the active metabolic compound responsible for the growth promotion were patented for commercial use (Hirt et al., 2018, 2019). These studies provide new solutions in performing agriculture with saline water irrigation.

Overall, desert plants represent rich niches and an ideal source for the isolation of novel PGPR, these PGPR are known to be equipped with unique traits that can help the plants to withstand the harsh conditions imposed by the desert. All taken together, desert PGPR can work as bio-fertilizers to improve the plants growth and tolerance against many abiotic stress conditions (e.g., salinity, drought and nutrient deficient soils, etc.), as well as enhancing soil fertility in degraded soils regions. In addition, desert PGPR can work as biocontrol agents against plant pathogens (e.g., fungal infections), as well as a source for antibacterial compounds that could be implied medically against human pathogens. Therefore, desert PGPR could play a vital role in improving the quality of crop plants and productivity for a sustainable agriculture in the arid, semi-arid, and hyper arid regions of the world, and have enormous benefits for industrial and medical applications.

## Concluding Remarks

Deserts make up 33% of the land total area in the world and occupy every continent. Life in these regions is challenged by harsh environmental conditions of abiotic stresses such as extreme temperature fluctuations, high radiation, water scarcity, low nitrogen, organic matter, and soil salinity. The desert habitats limit plant growth and vegetation to shrubs and short grasses. However, by genetic selection over thousands of years, desert plants have acquired specific mechanisms to thrive on soils with extremely low water content, salinity, and high temperatures. Desert plants receive help from bacterial communities with specific features such as exhibiting higher expression of genes related to dormancy and osmoregulation, as well as lower expression of genes regulating nutrient cycling and catabolism. These features indicate that these bacterial communities have adapted to the harsh environments of deserts. Despite the extreme conditions, the soil of the deserts across the world harbor abundant and diverse microbial communities, from which the most ubiquitous bacterial phyla are Actinobacteria, Proteobacteria, Bacteroidetes, Cyanobacteria, and Firmicutes. Desert bacterial communities associated with desert plants rhizosphere, phyllosphere, and rhizosheath are mainly influenced by soil aridity, vegetation cover, the geographical location, as well as seasonal and climatic changes. Abiotic and biotic factors, including soil composition, depth, texture, pH, electrical conductivity, salinity and water availability, shape the taxonomic, and functional groups of the communities. The bacterial communities in the plant endosphere are determined by plant root exudates, the plant species, their genotypes and the plant compartments (Berg and Smalla, 2009; Ramawat, 2010; Fierer et al., 2012; Hacquard et al., 2015; Saad et al., 2020).

Plants and their microbial communities work together as a consortium and act as a single biological entity the “holobiont,” coevolving as a unit of selection in evolution and determining the “hologenome.” Hereby, the host plant establishes symbiotic relationships with a huge diversity of microbial communities, and the combined genomes of host and its microbial community are potentially transmitted with accuracy from one generation to another. Moreover, there is a cooperation between the host plant and the microbial communities with the aim of improved fitness in many ways of development, behavior, morphology, disease resistance, and physiology. Mutualist members of the holobiont such as bacteria and fungi can directly interact with the host plants thereby providing fundamental processes in exchange for a niche and nutrients. Nitrogen-fixing bacteria, mycorrhizal fungi and plant growth promoting rhizobacteria are famous mutualist examples (Zilber-Rosenberg and Rosenberg, 2008; Moran and Sloan, 2015).

Finally, desert plants display a perfect niche for isolating novel PGPR that are well adapted to extreme environments (salt, heat and drought, and high UV tolerant) and exploit them for their extraordinary capabilities as bio-fertilizers and bio-control agents against a wide range of abiotic and biotic stresses endangering agricultural ecosystems. Desert PGPR might be key to improve soil fertility and increase plant tolerance and crop productivity and human health. Moreover, comparative genomics of those new isolates will provide a new toolbox for better understanding adaptation and evolution of life in extreme environments, this will help not only in our planet earth but will be lesson to learn for habituating other planet, e.g., Planet Mars. We strongly believe that the second green revolution is looming from belowground of desert plants.

## Author Contributions

WA and MS collected the literature, prepared the figures, and drafted the manuscript. HH supervised and corrected the manuscript. All authors listed have approved it for publication.

## Conflict of Interest

The authors declare that the research was conducted in the absence of any commercial or financial relationships that could be construed as a potential conflict of interest.

## Footnotes

1 http://www.darwin21.org
